# Association between physical activity and insulin resistance using the homeostatic model assessment for insulin resistance independent of waist circumference

**DOI:** 10.1038/s41598-022-10032-2

**Published:** 2022-04-09

**Authors:** Tae Kyung Yoo, Byeong Kil Oh, Mi Yeon Lee, Ki-Chul Sung

**Affiliations:** 1 Department of Medicine, MetroWest Medical Center, 115 Lincoln St, Framingham, MA 01702 USA; 2 Division of Cardiology, Department of Internal Medicine, Kangbuk Samsung Hospital, Sungkyunkwan University School of Medicine, 29 Saemunan-ro, Jongno-gu, Seoul, 03181 Republic of Korea; 3 Division of Biostatistics, Department of R&D Management, Kangbuk Samsung Hospital, Sungkyunkwan University School of Medicine, Seoul, Republic of Korea

**Keywords:** Biomarkers, Endocrinology, Health care

## Abstract

Only a few studies have evaluated the relationship between physical activity (PA) and Homeostatic model assessment for insulin resistance (HOMA-IR). Therefore, we aimed to analyze the association between HOMA-IR and PA. We included 280,194 Korean without diabetes who underwent health examinations. The short form of the International Physical Activity Questionnaire was completed. PA level was divided into sedentary, mild PA, and health-enhancing PA (HEPA). The HOMA-IR levels were calculated. Confounding factors including waist circumference were adjusted. The median follow-up duration was 4.13 years. A significant inverse relationship was observed between PA level and HOMA-IR (*p* < 0.001). Compared with the sedentary group, HOMA-IR was lower in the HEPA group (*p* < 0.001), even when HEPA group decreased PA level over time (*p* < 0.001). Mild PA (*p* < 0.001) or HEPA showed a lower risk of HOMA-IR progression (*p* < 0.001). Increasing PA or maintaining HEPA was significantly associated with a lower HOMA-IR (*p* < 0.001), HOMA-IR improvement (*p* < 0.001), and a lower risk of HOMA-IR progression (*p* < 0.001). Our findings support the inverse relationship between PA and HOMA-IR in a population without diabetes. PA might improve IR and prevent its progression among populations without diabetes, independent of the waist circumference.

## Introduction

Physical activity (PA) consists of movements using the skeletal muscles, which require the use of energy^1^. An irrefutable evidence supports the beneficial effects of PA^2^. It can decrease the morbidity and mortality due to cardiovascular disease (CVD), some types of cancers, obesity, fall risk, type 2 diabetes mellitus (DM), improve brain health, and reduce the all-cause mortality risk^1,3^. This benefit is not only observed in a single age group or population; virtually everyone can benefit from becoming more physically active^2^. Due to its proven effect, most international guidelines recommend that adults perform at least 150 min/week of moderate intensity or 75 min/week of vigorous-intensity PA^1,2^.

Insulin resistance (IR) is a common pathophysiological phenomenon and is defined as the inability of a known quantity of insulin to increase the glucose uptake and utilization^4,5^. IR has become increasingly prevalent at all ages, including overweight and sedentary middle-aged populations^6^. It is associated with metabolic syndrome^5^. In addition, it contributes to the development of associated metabolic derangements, such as type 2 DM, CVD, and non-alcoholic fatty liver disease (NAFLD)^7,8^. Previous clinical studies have reported that various IR-related diseases can be prevented by reducing IR^9,10^. Many studies have shown the effects of exercise on IR^5^. Based on these findings, physical exercise has been suggested to reverse IR and its associated conditions^11,12^.

The homeostasis model assessment-estimated IR (HOMA-IR) was used to estimate insulin sensitivity using the fasting plasma glucose and insulin concentrations^13^. Although the hyperinsulinemic-euglycemic glucose clamp test is considered the gold standard for measuring IR, its clinical applicability is limited as this procedure is labor intensive and costly^14,15^. Meanwhile, HOMA-IR is an accurate clinical and epidemiological tool that is used to describe the pathophysiology of diabetes^16^. It is also an easily obtainable, safe, less invasive, and less expensive method compared with the euglycemic clamp test, while its results are well correlated with those of the euglycemic clamp test^17,18^. Due to such strengths, many epidemiologic studies have implemented HOMA-IR to estimate the IR in participants^19^. To date, only a few studies have investigated the relationship between PA and IR expressed as HOMA-IR, but the quality of evidence is low^20^. In addition, this association has not been proven in a large population. Consequently, the extent to which the reported relationship can be applied to individuals remains unclear. Therefore, this study used the data of a large cohort to evaluate the relationship between PA and IR, expressed as HOMA-IR, which can be used in large-scale epidemiologic studies. In addition to the cross-sectional relationship, this study assessed the change of PA over time and its effect on HOMA-IR.

## Results

### Cohort description

The median follow-up duration was 4.13 years. The participants had a minimum of 2 to a maximum of 10 examinations during the follow-up period. The mean number of examinations that the participants had was 3.94 ± 1.84. Table 1 presents the participants’ baseline characteristics. Among the 280,194 participants, 49.2% (n = 137,830) were sedentary group, 35.1% (n = 98,309) were mild PA group and 15.7% (n = 44,055) were HEPA group. The mean age of the participants was 38.2 ± 7.7, and the median HOMA-IR was 1.18 (0.78–1.74). All the variables showed a significant difference between each PA group.

**Table 1:** Baseline characteristics.

	Sedentary	Mild	HEPA	*p* value
Number	137,830 (49.2)	98,309 (35.1)	44,055 (15.7)	
Age	37.6 ± 7.2	38.3 ± 7.7	39.4 ± 8.7	< 0.001
**Sex %**				< 0.001
Male	67,703 (49.12)	61,451 (62.51)	26,882 (61.02)	
Female	70,127 (50.88)	36,858 (37.49)	17,173 (38.98)	
Current smoker, %	27,095 (19.66)	21,505 (21.87)	8,843 (20.07)	< 0.001
Alcohol intake (g/day)	6 (2–14)	6 (3–17)	7 (3–21)	< 0.001
High alcohol intake, %	20,063 (14.56)	15,270 (15.53)	8,351 (18.96)	< 0.001
BMI, kg/m^2^	23.0 ± 3.4	23.4 ± 3.2	23.5 ± 3.1	< 0.001
Waist, cm	80.6 ± 9.8	81.7 ± 9.4	81.7 ± 9	< 0.001
Higher education, %	104,140 (75.56)	76,130 (77.44)	30,850 (70.03)	< 0.001
SBP, mmHg	107.7 ± 12.8	109.8 ± 12.8	110.8 ± 12.9	< 0.001
HOMA-IR	1.2 (0.8–1.8)	1.2 (0.8–1.7)	1.1 (0.7–1.6)	< 0.001

Numbers in the table are mean ± standard deviation, median (interquartile range), or (percentages).

High alcohol intake defined as > 30 g/day for men and > 20 g/day for women; higher education defined as college graduate or higher.

IPQ, International Physical Activity Questionnaire; BMI, body mass index; SBP, systolic blood pressure; HOMA-IR, homeostasis model assessment of insulin resistance.

### Sex-stratified associations of PA with HOMA-IR

Table 2 shows the sex-stratified associations of PA with HOMA-IR. In a multivariable model, the mild PA group and HEPA group showed lower HOMA-IR compared with the sedentary group (sedentary group, ref; mild, estimate: 0.96, Confidence Interval (CI) 0.96–0.96, *p* < 0.001; HEPA, estimate: 0.9, CI 0.89–0.90, *p* < 0.001). In the female group, the same trend was observed in the multivariable model (mild, estimate: 0.97, CI 0.96–0.97, *p* < 0.001; HEPA, estimate 0.91, CI 0.90–0.92, *p* < 0.001). In the male group, the same trend was observed and remained significant (multivariable model = mild, estimate: 0.96, CI 0.95–0.96, *p* < 0.001; HEPA, estimate: 0.9, CI 0.89–0.9, *p* < 0.001).

**Table 2:** Sex stratified associations of PA with HOMA-IR.

	Crude model		Age-sex adjusted		Multivariable model	
	Estimate (95% CI)	*p* value	Estimate (95% CI)	*p* value	Estimate (95% CI)	*p* value
**Total**						
Sedentary	1 (reference)		1 (reference)		1 (reference)	
Mild	0.96 (0.95–0.96)	< 0.001	0.95 (0.94–0.95)	< 0.001	0.96 (0.96–0.96)	< 0.001
HEPA	0.89 (0.88–0.89)	< 0.001	0.88 (0.87–0.88)	< 0.001	0.9 (0.89–0.9)	< 0.001
**Female**						
Sedentary	1 (reference)		1 (reference)		1 (reference)	
Mild	0.96 (0.95–0.96)	< 0.001	0.95 (0.95–0.96)	< 0.001	0.97 (0.96–0.97)	< 0.001
HEPA	0.91 (0.9–0.91)	< 0.001	0.9 (0.89–0.91)	< 0.001	0.91 (0.9–0.92)	< 0.001
**Male**						
Sedentary	1 (reference)		1 (reference)		1 (reference)	
Mild	0.95 (0.94–0.95)	< 0.001	0.95 (0.94–0.95)	< 0.001	0.96 (0.95–0.96)	< 0.001
HEPA	0.87 (0.86–0.87)	< 0.001	0.87 (0.86–0.87)	< 0.001	0.9 (0.89–0.9)	< 0.001

Estimated value represents exponential coefficient [exp (beta)] from the linear mixed model with random effects.

Sedentary, less than 600MET-minutes per week of physical activity; Mild physical activity, 600 MET-minutes per week; Health-enhancing physical activity, HEPA: 3000 MET-minutes per week; CI, confidence interval; HOMA-IR, homeostasis model assessment-estimated IR; PA, physical activity.

Multivariable model: Adjusted for age, sex, systolic blood pressure, smoking, level of education, waist circumference, alcohol intake (for each sex stratified analysis, sex was not adjusted).

### Sex-stratified associations of PA with HOMA-IR according to the changes in PA level

Table 3 shows the sex-stratified associations of PA with HOMA-IR according to the changes in PA levels. The participants were divided into four groups based on the changes in the PA level: sedentary and mild PA level (SM) to SM (reference), HEPA to SM, SM to HEPA, and HEPA to HEPA. Compared with the SM to SM group, the other three groups showed significantly decreased HOMA-IR levels in the multivariable model (HEPA to SM group = estimate: 0.94, CI 0.93–0.94, *p* < 0.001; SM to HEPA group = estimate: 0.93, CI 0.93–0.94, *p* < 0.001; HEPA to HEPA group = estimate: 0.86, CI 0.85–0.87, *p* < 0.001).

**Table 3:** Sex-stratified associations of PA with HOMA-IR according to the changes in PA level.

	Crude model		Age-sex adjusted		Multivariable model	
	Estimate (95% CI)	*p* value	Estimate (95% CI)	*p* value	Estimate (95% CI)	*p* value
**Total**						
SM → SM	1 (reference)		1 (reference)		1 (reference)	
H → SM	0.96 (0.95–0.96)	< 0.001	0.95 (0.94–0.95)	< 0.001	0.94 (0.93–0.94)	< 0.001
SM → H	0.96 (0.95–0.97)	< 0.001	0.94 (0.93–0.95)	< 0.001	0.93 (0.93–0.94)	< 0.001
HEPA → HEPA	0.88 (0.86–0.88)	< 0.001	0.85 (0.84–0.85)	< 0.001	0.86 (0.85–0.87)	< 0.001
**Female**						
SM → SM	1 (reference)		1 (reference)		1 (reference)	
HEPA → SM	0.98 (0.96–0.99)	< 0.001	0.97 (0.96–0.98)	< 0.001	0.95 (0.94–0.96)	< 0.001
SM → HEPA	0.98 (0.97–0.99)	< 0.001	0.97 (0.96–0.98)	< 0.001	0.95 (0.94–0.96)	< 0.001
HEPA → HEPA	0.89 (0.88–0.9)	< 0.001	0.88 (0.86–0.89)	< 0.001	0.88 (0.86–0.89)	< 0.001
**Male**						
SM → SM	1 (reference)		1 (reference)		1 (reference)	
HEPA → SM	0.93 (0.92–0.94)	< 0.001	0.93 (0.92–0.94)	< 0.001	0.93 (0.92–0.94)	< 0.001
SM → HEPA	0.93 (0.92–0.93)	< 0.001	0.92 (0.91–0.93)	< 0.001	0.93 (0.92–0.93)	< 0.001
HEPA → HEPA	0.84 (0.83–0.85)	< 0.001	0.83 (0.82–0.84)	< 0.001	0.86 (0.85–0.86)	< 0.001

Estimated value represents exponential coefficient [exp (beta)] from the linear mixed model with random effects.

S, Sedentary, less than 600MET-minutes per week of physical activity; M, Mild physical activity , 600 MET-minutes per week; HEPA, Health-enhancing physical activity, : 3000 MET-minutes per week; CI, confidence interval; HOMA-IR, homeostasis model assessment-estimated IR; PA, physical activity.

Multivariable Model : Adjusted for age, sex, systolic blood pressure, smoking, level of education, waist circumference, alcohol intake. (for each sex stratified analysis, sex was not adjusted).

The association was assessed for each sex. In the female group, the multivariable model (HEPA to SM group = estimate: 0.95, CI 0.94–0.96, *p* < 0.001; SM to HEPA group = estimate: 0.95, CI 0.94–0.96, *p* < 0.001; HEPA to HEPA group = estimate: 0.88, CI 0.86–0.89, *p* < 0.001) showed the same trend.

The male group also showed the same trend in the multivariable model (HEPA to SM group = estimate: 0.93, CI 0.92–0.94, *p* < 0.001; SM to HEPA group = estimate: 0.93, CI 0.92–0.93, *p* < 0.001; HEPA to HEPA group = estimate: 0.86, CI 0.85–0.86, *p* < 0.001).

### Associations of PA with the change of HOMA-IR level

As shown in Table 4, the participants were divided into two groups; baseline HOMA-IR ≥ 2.2 group (n = 38,950) and baseline HOMA-IR < 2.2 group (n = 241,244). In each group, the association between PA and the change of HOMA-IR level (improvement or progression) was investigated.

**Table 4:** Associations of PA with the change of HOMA-IR level.

	Person year	Incident cases	Incidence rate (per 100 PY)	Crude model		Age-sex adjusted		Multivariable model		Time dependent model*	
				HR (95% CI)	*p* value	HR (95% CI)	*p* value	HR (95% CI)	*p* value	HR (95% CI)	*p* value
**Incidence of improvement**^**a**^											
Sedentary	58,762.9	11,697	19.9 (19.6–20.3)	1 (reference)		1 (reference)		1 (reference)		1 (reference)	
Mild	35,843.7	7299	20.4 (19.9–20.8)	1.04 (1.01–1.07)	0.018	1.05 (1.02–1.09)	< 0.001	1.02 (0.99–1.05)	0.176	1.02 (0.99–1.06)	0.120
HEPA	12,934.5	2677	20.7 (19.9–21.5)	1.05 (1.01–1.10)	0.016	1.05 (1.01–1.10)	0.018	1.02 (0.98–1.07)	0.292	1.03 (0.99–1.08)	0.166
**Incidence of progression**^**b**^											
Sedentary	452,945.3	32,528	7.2 (7.1–7.3)	1 (reference)		1 (reference)		1 (reference)		1 (reference)	
Mild	311,672.6	22,447	7.2 (7.1–7.3)	1.00 (0.99–1.02)	0.61	0.93 (0.92–0.95)	< 0.001	0.96 (0.94–0.98)	< 0.001	0.97 (0.95–0.99)	0.001
HEPA	141,421.9	9468	6.7 (6.6–6.8)	0.94 (0.92–0.96)	< 0.001	0.89 (0.87–0.91)	< 0.001	0.94 (0.92–0.96)	< 0.001	0.93 (0.91–0.95)	< 0.001

Sedentary, less than 600MET-minutes per week of physical activity; Mild physical activity, 600 MET-minutes per week; HEPA, Health-enhancing physical activity : 3000 MET-minutes per week; CI, confidence interval; HOMA-IR, homeostasis model assessment-estimated IR; HR, hazard ratio; PA, physical activity.

Multivariable model : Adjusted for age, sex, systolic blood pressure, smoking, level of education, waist circumference, change of waist circumference (difference between waist circumference in last follow up and baseline), alcohol intake, baseline HOMA-IR.

Time dependent model: Adjusted for age, sex, systolic blood pressure, smoking, level of education, waist circumference, baseline HOMA-IR, alcohol intake (waist circumference as time-varying covariates).

^a^Incidence of improvement; Analyzed among the participants who had HOMA-IR ≥ 2.2 at baseline (n = 38,950).

^b^Incidence of progression; Analyzed among the participants who had HOMA-IR < 2.2 at baseline (n = 241,244).

#### Baseline HOMA-IR ≥ 2.2 group; association between PA and HOMA-IR improvement

In the baseline HOMA-IR ≥ 2.2 group, in the crude model, mild PA and HEPA groups were more likely to have HOMA-IR improvement than the sedentary group (sedentary group = ref; mild PA = hazard ratio (HR): 1.04, CI 1.01–1.07, *p* = 0.018; HEPA group = HR: 1.05, CI 1.01–1.10, *p* = 0.016). However, in the multivariable model (mild PA = HR: 1.02, CI 0.99–1.05, *p* = 0.176; HEPA group = HR: 1.02, CI 0.98–1.07, *p* = 0.292) and time-dependent model (mild PA = HR: 1.02, CI 0.99–1.06, *p* = 0.120; HEPA group = HR: 1.03, CI 0.99–1.08, *p* = 0.166), no significant difference was observed between all groups.

#### Baseline HOMA-IR < 2.2 group; association between PA and HOMA-IR progression

In the baseline HOMA-IR < 2.2 group, in the crude model, the HEPA group showed a lower HOMA-IR progression risk than the sedentary and mild PA group, while no significant difference was found between the sedentary and mild PA groups (sedentary group = ref; mild PA = HR: 1.00, CI 0.99–1.02, *p* = 0.61; HEPA group = HR: 0.94, CI 0.92–0.96, *p* < 0.001). After the confounding factor adjustment, both mild PA and HEPA group showed lower HOMA-IR progression risk than the sedentary group in the multivariable model (mild PA = HR: 0.96, CI 0.94–0.98, *p* < 0.001; HEPA group = HR: 0.94, CI 0.92–0.96, *p* < 0.001) and time-dependent model (mild PA = HR: 0.97, CI 0.95–0.99, *p* = 0.001; HEPA group = HR: 0.93, CI 0.91–0.95, *p* < 0.001).

### Associations of change in the PA with change in HOMA-IR

Table 5 shows the associations between changes in PA level and changes in HOMA-IR level. The participants were divided into two groups; baseline HOMA-IR ≥ 2.2 group (n = 38,950) and baseline HOMA-IR < 2.2 group (n = 241,244). In each groups, the association between changes in PA level (SM to SM, HEPA to SM, SM to HEPA, HEPA to HEPA) and changes in HOMA-IR level (improvement, progression) were investigated.

**Table 5:** Associations of change in the PA with change in HOMA-IR.

	Person year	Incident cases	Incidence Rate (per 100 PY)	Crude model		Age-sex adjusted		Multivariable model		Time dependent model	
				HR (95% CI)	*p* value	HR (95% CI)	*p* value	HR (95% CI)	*p* value	HR (95% CI)	*p* value
**Incidence of improvement**^**a**^											
SM → SM	82,261.7	16,254	19.8 (19.5–20.1)	1 (reference)		1 (reference)		1 (reference)		1 (reference)	
HEPA → SM	9,032.7	1649	18.3 (17.4–19.2)	0.92 (0.88–0.97)	0.002	0.92 (0.87–0.97)	0.001	0.97 (0.91–1.04)	0.436	0.93 (0.89–0.98)	0.010
SM → HEPA	11,866.8	2758	23.2 (22.4–24.1)	1.18 (1.13–1.23)	< 0.001	1.18 (1.13–1.23)	< 0.001	1.11 (1.06–1.16)	< 0.001	1.21 (1.16–1.26)	< 0.001
HEPA → HEPA	4,379.8	1012	23.1 (21.7–24.6)	1.19 (1.11–1.26)	< 0.001	1.18 (1.10–1.25)	< 0.001	1.11 (1.03–1.21)	0.011	1.15 (1.07–1.23)	< 0.001
**Incidence of progression**^**b**^											
SM → SM	662,018.9	48,164	7.3 (7.2–7.3)	1 (reference)		1 (reference)		1 (reference)		1 (reference)	
HEPA → SM	95,764.2	7125	7.4 (7.3–7.6)	1.01 (0.98–1.03)	0.587	0.99 (0.97–1.02)	0.466	0.98 (0.95–1.01)	0.151	1.03 (1.001–1.05)	0.048
SM → HEPA	94,329	6082	6.5 (6.3–6.6)	0.87 (0.85–0.89)	< 0.001	0.85 (0.83–0.87)	< 0.001	0.87 (0.84–0.89)	< 0.001	0.80 (0.78–0.83)	< 0.001
HEPA → HEPA	53,927.8	3072	5.7 (5.5–5.9)	0.78 (0.75–0.81)	< 0.001	0.75 (0.73–0.78)	< 0.001	0.81 (0.78–0.85)	< 0.001	0.81 (0.78–0.84)	< 0.001

Multivariable model : Adjusted for age, sex, systolic blood pressure, smoking, level of education, waist circumference, change of waist circumference (difference between waist circumference in last follow up and baseline), alcohol intake, baseline HOMA-IR.

Time dependent model: Adjusted for age, sex, systolic blood pressure, smoking, level of education, waist circumference, baseline HOMA-IR, alcohol intake (waist circumference as time-varying covariate).

S, Sedentary, less than 600MET-minutes per week of physical activity; M, Mild physical activity, 600 MET-minutes per week; HEPA, health-enhancing physical activity : 3000 MET-minutes per week; CI, confidence interval; HOMA-IR, homeostasis model assessment-estimated IR; HR, hazard ratio; PA, physical activity.

^a^Incidence of improvement; Analyzed among the participants who had HOMA-IR ≥ 2.2 at baseline (n = 38,950).

^b^Incidence of progression; Analyzed among the participants who had HOMA-IR < 2.2 at baseline (n = 241,244).

#### Baseline HOMA-IR ≥ 2.2 group; association between change in the PA and HOMA-IR improvement

In the baseline HOMA-IR ≥ 2.2 group, the participants whose PA level changed from HEPA to SM group was less likely to have HOMA-IR improvement in the crude model, and time dependent model, compared with the SM to SM group (crude = HR: 0.92, CI 0.88–0.97, *p* = 0.002; time dependent, HR: 0.93, CI 0.89–0.98, *p* = 0.010). The SM to HEPA group (multivariable model = HR: 1.11, CI 1.06–1.16, *p* < 0.001; time-dependent model = HR: 1.21, CI 1.16–1.26, *p* < 0.001) and HEPA to HEPA group (multivariable model = HR: 1.11, CI 1.03–1.21, *p* = 0.011; time-dependent model = HR: 1.15, CI 1.07–1.23, *p* < 0.001) were associated with HOMA-IR improvement in all models.

#### Baseline HOMA-IR < 2.2 group; association between change in the PA and HOMA-IR progression

In the baseline HOMA-IR < 2.2 group, the SM to HEPA group (multi-variable = HR: 0.87, CI 0.84–0.89, *p* < 0.001; time-dependent, HR: 0.80, CI 0.78–0.83, *p* < 0.001) and HEPA to HEPA group (multi-variable = HR: 0.81, CI 0.78–0.85, *p* < 0.001; time-dependent = HR: 0.81, CI 0.78–0.84, *p* < 0.001) were associated with a lower risk of HOMA-IR progression in all models. The HEPA to SM group were associated with a higher risk of HOMA-IR progression in the time-dependent model (HR: 1.03, CI 1.001–1.05, *p* = 0.048).

## Discussion

Our results showed that there was a significant inverse relationship between PA level and HOMA-IR, a marker of IR. Second, compared with the sedentary group, HOMA-IR was lower even if the PA level in the HEPA group was decreased over time. Third, mild PA and HEPA showed a lower risk of HOMA-IR progression. Fourth, increasing the PA level or maintaining HEPA levels was significantly associated with lower HOMA-IR level. Lastly, the increasing PA or maintaining HEPA level was associated with HOMA-IR improvement and a lower risk of HOMA-IR progression.

As a well-known fact, type 2 DM develops as a result of IR and is associated with metabolic abnormalities^4^. In addition, diabetes medications including metformin, glimepiride, and SGLT2 inhibitors can affect the HOMA-IR levels^21,22^. Previous studies that assessed the relationship between PA and HOMA-IR were limited due to their small sample sizes^23,24^, were not adjusted for waist circumference as a confounding factor^25,26^, were conducted in pregnant women^24^, did not exclude the diabetes population^26^, or were conducted in type 2 DM patients^23^. Owing to these limitations, the quality of evidence is relatively low^20^. By excluding participants with DM, incorporating a large number of cohorts, and conducting extensive adjustment for confounding factors, our study provided more reliable results than the previous studies.

Our study suggested the possible lingering effect of increased PA on IR, even after the individual’s PA level was decreased. This finding can be explained by the cumulative effect of exercise on IR and insulin sensitivity^27^. A previous study including 346 men and 455 women from the RISC study showed that the total amount of and accumulated number of PAs performed were the determinants of insulin sensitivity^27^. Even when the physically active participants’ level of activity decreases, they still have a higher amount of total accumulated PA than the continuously sedentary population. This higher accumulated PA time in participants with decreased PA level from HEPA to SM might have led to the reduction in the HOMA-IR level.

In addition, our findings suggest that PA might have a greater impact on attenuating HOMA-IR progression than resolving the pre-existing IR. This finding supports the pre-existing notion of performing PAs as a method to prevent or delay the development of type 2 DM, which results from IR and loss of insulin secretion^28,29^. Furthermore, increasing the PA level or maintaining a high level of PA is associated with HOMA-IR improvement and prevention of HOMA-IR progression, while decreasing the PA level makes individuals more susceptible to HOMA-IR progression and decreases the likelihood of HOMA-IR improvement. Overall, our findings consistently support the beneficial effects of PA on IR^11,29^.

PA has diverse influences on IR and glucose metabolism through acute changes that cause contraction-mediated glucose uptake through glucose transporter 4, and chronic adaptations causing insulin-stimulated glucose uptake^6,30,31^. Although numerous studies support the beneficial effect of PA on IR, it remains unclear whether the effect of exercise is due to the decrease in waist circumference or whether it is the effect of exercise itself^32,33^. A cross-sectional study conducted in 6,500 adults in the United States showed that PA is associated with IR^33^. However, this relationship disappeared after adjusting for differences in waist circumference, suggesting that visceral fat, expressed as waist circumference^34^, mediates the relationship between PA level and HOMA-IR^33^. Meanwhile, another study conducted in a Canadian population showed an independent association between PA and insulin sensitivity in men after adjusting for waist circumference^35^. Our study results support the finding that PA per se has a direct association with IR. However, further prospective studies are warranted to verify the relationship between PA, visceral fat, and IR.

Our study is unique as it was conducted in a large number of participants (n = 280,194), including both men (n = 156,036) and women (n = 124,158). Participants who were newly diagnosed with diabetes during the health examination and those previously diagnosed with diabetes with or without medical treatment were excluded, which made our results more reliable. A robust adjustment for confounding factors was performed, and a time-dependent analysis of waist circumference, a strong independent risk factor for IR, was carried out to verify the independent association between PA and IR, expressed as HOMA-IR^36^. Moreover, the dynamic relationship between the change in PA level over time and HOMA-IR was assessed. To the best of our knowledge, this was the first study to assess such associations. In addition, this was the first study to assess the association between changes in PA level and HOMA-IR trend over time.

Despite these strengths, our study has several limitations. First, this study only included Korean individuals. Second, a self-report form (IPAQ) was used to assess the PA level since this tool is useful for evaluating a large cohort^37^. Although the IPAQ is a valid form to assess the PA level of an individual^37,38^, self-reporting and recall bias can occur^30^. Third, our study participants were young (mean age: 38.2 ± 7.7) and highly educated population (higher education = 75.4%). Age and educational attainment were associated with IR^39,40^. To overcome these limitations, we adjusted for age and education as confounding factors. In addition, the relatively young age of our study participants can highlight the relationship between PA and IR in a relatively young population. However, future prospective studies incorporating diverse races and populations are warranted to verify our results.

In conclusion, our study showed that PA level has an inverse relationship with IR, expressed as HOMA-IR. The positive effect of a high level of PA lingered even when the level of activity decreased over time. In addition, PA level might slow the progression of IR among populations without underlying IR, independent of the waist circumference and BMI status. Increasing the level of PA or maintaining HEPA can slow the progression of IR and improve IR. Our findings support the beneficial effect of PA on IR, which is associated with type 2 DM, hypertension, and dyslipidemia^5^.

## Methods

### Study population

The Kangbuk Samsung Health Study (KSHS) data were used in the study. The KSHS is an ongoing cohort study conducted in a Korean population aged 18 years and older who underwent comprehensive health examinations at one of the two total healthcare centers of Kangbuk Samsung Hospital in Seoul and Suwon, South Korea. In South Korea, all employees are required to undergo annual or biennial health screening examinations in accordance with the Industrial Safety and Health Law. More than 80% of the participants in the current study were either employees or spouses of employees of various companies and local government organizations. The remaining participants underwent medical checkups of their own accord.

**Figure 1:**
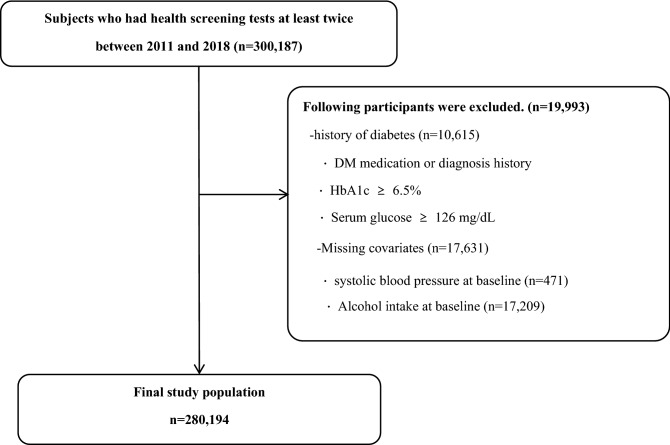
Flow diagram for study participants.

In the KSHS, 300,187 individuals who underwent a comprehensive health examination at least twice between 2011 and 2018 were initially included. Those who met the following criteria were excluded from the analysis: participants with DM at baseline (determined based on the following factors: self-reported diabetes, use of anti-glycemic medications, or previously diagnosed with DM, as indicated in the medical records), a fasting plasma glucose level of ≥ 126 mg/dL, and a hemoglobin A1c (HbA1c) level of ≥ 6.5%) (n = 10,615); individuals with missing covariates (systolic blood pressure [SBP], n = 574; alcohol intake, n = 17,209) were excluded. Overall, 280,194 participants were included in the final analysis (Fig. 1). This study was approved by the Institutional Review Board (IRB) of Kangbuk Samsung Hospital (IRB no: 2015-12-004-017). Informed consent was waived by the IRB of Kangbuk Samsung Hospital because anonymized and de-identified data were used in the analysis. All study methods were conducted in accordance with relevant guidelines and regulations.

### Measurement

During health screening, the self-administered questionnaires were used to collect the demographic data, medical history, socioeconomic history including smoking status and alcohol intake, educational background, and level of PA. Alcohol intakes of > 30 g/day for men and > 20 g/day for women were defined as high alcohol intake^41,42^; higher education was defined as college graduate or a higher level of education. The National Health Interview Survey criteria were used to define the smoking status. Current smokers were defined as those who smoked more than 5 packs (more than 100 cigarettes) in their lifetime and currently smoking at the time of the interview. A former smoker was defined as a person who had smoked more than 100 cigarettes in their lifetime but who had quit smoking at the time of the interview^43^.

The self-administered form of the Korean version of the International Physical Activity Questionnaire (IPAQ) was used to validate the PA levels^38^. In the questionnaire, participants were instructed to record the frequency and duration of PA over the past 7 days. All participants indicated the frequency (0–7 days/week) of every moderate or vigorous PA performed. PAs that lasted more than 10 min were included in the count. The duration of PA was recorded on a daily basis (min/day). In the same way, the time that the participants performed walking and other physical movements, including transportation, house chores, and working and leisure activities, were recorded (0–7 days/week and minutes/day). The total physical inactivity time was assessed using the following question: “During the last 7 days, how much time did you spend sitting or lying per each day?” Physical inactivity was defined as all activities performed while sitting or lying down. Strength exercises such as push-ups were counted separately based on the number of times per week. The participants were classified into three categories: sedentary, mild PA (600 metabolic equivalent of task [MET]-minutes per week), and health-enhancing PA (3,000 MET-minutes per week)^38^.

Anthropometric measurements (height, weight, systolic blood pressure, and diastolic blood pressure) were performed by trained medical staff. During the measurements, the participants wore a lightweight hospital gown (< 0.1 kg) without shoes. Body mass index (BMI) was calculated as weight divided by height in meters squared (kg/m^2^). Blood pressure (BP) was measured after a period of rest in a sitting position. During the BP measurement, the arm was positioned at the heart level, and an automated oscillometric device (53,000, Welch Allyn, New York, USA) was used. Blood biochemical samples were collected after fasting for > 10 h. The blood samples were analyzed by the Laboratory Medicine Department at Kangbuk Samsung Hospital, which has been accredited by the Korean Association of Quality Assurance for Clinical Laboratories and the Korean Society of Laboratory Medicine.

### HOMA-IR

The following formula was used to calculate the HOMA-IR: fasting plasma insulin (μU/ml) × fasting plasma glucose (mg/dl)/405^44^. The HOMA-IR value of 2.2 was assigned as the cut-off value, following the cut-off value in the Korean population^45^. Participants were divided into HOMA-IR < 2.2 or HOMA-IR ≥ 2.2 groups based on their baseline HOMA-IR value. In the HOMA-IR ≥ 2.2 group, the change of HOMA-IR value from ≥ 2.2 to < 2.2 during the follow-up period was defined as the HOMA-IR improvement. In the HOMA-IR < 2.2 group, the change of HOMA values from < 2.2 to ≥ 2.2 during the follow up period was defined as the HOMA-IR progression.

### Statistical analysis

All statistical analyses were conducted using STATA version 16.1 (StataCorp LP, College Station, TX, USA). Continuous variables were expressed as mean ± standard deviation (SD) or median [interquartile range (IQR)], based on the distribution.

Student’s t-test or the Mann–Whitney test was used to compare continuous variables between the two groups. Analysis of variance or Kruskal–Wallis test was used to compare multiple groups. The HOMA-IR with a right-skewed distribution was logarithmically transformed. A generalized mixed model with random effects (of individual and error) was performed to assess the longitudinal associations between HOMA-IR and PA category. The slope was estimated using the exponential coefficients and 95% CIs in the model. The HRs and 95% CIs for each improvement and progression of HOMA-IR changes according to the PA category were estimated using the Cox proportional hazards model. The multivariable model was adjusted for age, sex, SBP, smoking status (never, past, or current), educational level (< college education or ≥ college education), waist circumference, baseline HOMA-IR, and waist circumference change. A parametric proportional hazard model, including waist circumference as a time-varying covariate, was additionally implemented as a time-dependent model. For the time-varying covariate (waist circumference) and HOMA-IR level, all the data during the follow-up period were used for the analysis. For the PA, the data at baseline and the data at the end of the follow-up period (last follow-up) were used for the analysis. For all other variables, the data at baseline was used for the analysis. Statistical significance was defined as a two-tailed *p* value of < 0.05.

### Ethics approval and consent to participate

This study was approved by the Institutional Review Board (IRB) of Kangbuk Samsung Hospital (IRB no: 2015-12-004-017). Informed consent was waived by the IRB of Kangbuk Samsung Hospital because anonymized and de-identified data were used in the analysis.

### Consent for publication

All authors gave full consent for publication.

## Data Availability

All data generated or analyzed during this study are included in this published article.
